# Binocular advantages in reading revisited: attenuating effects of individual horizontal heterophoria

**DOI:** 10.16910/jemr.12.4.10

**Published:** 2019-12-09

**Authors:** Stephanie Jainta, Joëlle Joss

**Affiliations:** Institute of Optometry, University of Applied Sciences, Northwestern Switzerland

**Keywords:** binocular coordination, reading, binocular advantages, eye movement, heterophoria, optometric tests

## Abstract

Reading with two eyes necessitates efficient processes of binocular vision, which provide a single percept of the text. These processes come with a binocular advantage: binocular reading shows shorter average fixation durations and sentence reading times when compared to monocular reading. A couple of years ago, we showed for a small sample (N=13) that binocular advantages critically relate to the individual heterophoria (the resting state of vergence). In the present, large-scale replication we collected binocular eye movements (Eyelink II) for 94 participants who read 20 sentences monocularly and 20 sentences binocularly. Further, individual heterophorias were determined using three different optometric standards: objective eye tracking (EyeLink II at 60 cm), Maddox wing test (at 30 cm) and measures following the “Guidelines for the application of the **M**easuring and **C**orrecting Methodology after H.-J. **H**aase” (MCH; at 6 m). Binocular eye movements showed typical pattern and we replicated (1) binocular advantages of about 25 ms for average fixation durations and (2) a reduction in binocular advantages when heterophoria increased – but only when heterophoria was identified by EyeLink II or Maddox wing measures; MCH measures of heterophoria did not affect binocular advantages in reading. For large heterophorias binocular reading even turned into a disadvantage. Implications for effect estimations and optometric testing will be discussed.

## Introduction

Reading with two eyes necessitates efficient processes of binocular vision, which provide a stable, single percept of the text while the eyes perform a sequence of saccades from one word to the next. These fusion processes also come with a binocular advantage: binocular reading shows shorter fixation durations and sentence reading times when directly compared to monocular reading [1–3]. The literature on binocular vision suggests that such binocular advantages in reading might be purely induced by differences at low levels of visual processing and directly relate to summative effects that arise when two input signals are combined during sensory fusion [4–10]. In other words, the combined signal from the two visual receptors provides a richer source of information in relation to detection of, or discriminating between, visual features.

Moreover, we showed recently, that binocular advantages in reading go far beyond simple signal summation benefits: under binocular reading conditions, lexical identification was enabled to such an extent that word frequency effects (shorter fixations for more familiar words) emerged during the very first fixation on a binocularly fixated word even when parafoveal preview of that word was monocular [11]. However, these word frequency effects were smaller compared to those that occurred for binocular reading. Critically, we also demonstrated that under monocular viewing conditions, lexical identification was inhibited to such a degree that the frequency effect was not present.

Remember, however, that monocular vision is generally an atypical viewing condition (for most people without binocular vision problems) and it typically comes with higher visual thresholds for luminance and contrast, for example [4–7]. Recently, Johansson et al. [2] varied the contrast of monocular and binocular text presentations and showed that when contrasts are lowered, reading speed decreases and fixation durations increase. More importantly, binocular advantages in reading increased with reduced contrast of the presented text: the lower the contrast (down to 10%), the longer (up to 20%) the fixation durations in monocular reading compared to binocular reading. Thus, besides an overall slowing of binocular reading when text contrast was reduced [2, 12–14], variation of text contrast also impacts on the extent to which participants benefit from binocular vision during reading [2].

Furthermore, in Jainta and Jaschinski [15], we showed that binocular advantages (like shorter first fixation times in reading) relate to individual aspects of motor fusion (i.e. horizontal heterophoria). Such results are in agreement with reports of binocular advantages as a consequence (and summation) of several visual functions [5–7, 16]. Remember that the individual horizontal heterophoria reflects the vergence angle that results from proximal aspects, tonic vergence and accommodation [8, 9, 17]: when one eye is occluded, the vergence eye movement system adopts a resting position (called horizontal heterophoria or dissociated phoria); if the eyes remain aligned relative to the stimulus, that is, no deviation of the occluded eye is observed, this is called an orthophoria. If the occluded eye is moving temporally, the resulting uncrossed vergence angle (relative to the target’s viewing distance) is called exophoria. If the occluded eye moves nasally, i.e. creating a vergence angle with crossed visual axes relative to the targets viewing distance, this is called esophoria. Individuals differ in their horizontal heterophoria and it adapts to different viewing conditions [17–20] and impacts on preprogrammed aspects of vergence adjustments [21–23].

Nevertheless, the impact of horizontal heterophoria on binocular advantages in reading was reported for 13 participants and small heterophorias (range: 0 to 3 degrees exophoria) only [15]. Recently, a study addressing the reading performance for 16 participants when heterophoria (horizontal and vertical) was induced by prismatic lenses showed no obvious changes in reading behavior [24]. Thus, the first and obvious aim of this present study was to initiate a large-scale replication - including broader heterophoria ranges - to allow for reliable effect estimations. Further, clinically, in optometric testing for example, different methods are used to characterize individual horizontal heterophoria. There is no data yet characterizing differential impacts of different horizontal heterophoria measures on binocular advantages in reading. Note that, horizontal and vertical vergence adjustments during reading show quite different characteristics, and this dissociation is directly related to the functional role of vergence adjustments: vertical fusion – and vertical vergence – subserves the maintenance of a single percept and stereopsis by keeping the eyes in register and allowing for horizontal fusional processes to successfully operate over a vertically aligned input [25, 26]. Therefore, even though vertical heterophoria might be disruptive for reading (see, for example, Quercia et al. [27]; but Dysli et al. [24]), we will focus on horizontal heterophoria throughout this study.

Very generally, reported horizontal heterophoria tests can be classified as (1) objective methods using an eye tracker and (2) subjective methods, which typically rely on the participant’s perceptions. When considering objective measurements with an eye tracking device, heterophoria is reported for dynamic tasks, such as reading a text [15] or static tasks, such as centrally fixating a single target (a cross, line or dot; Han, Guo et al. [28]). In all cases, the fixation target is presented to one eye only (full dissociation of the two visual inputs), while the position of both eyes is being recorded. Further, the participant is completely absorbed by the task on hand and (almost) unaware of the measurements. In contrast, subjective measurements of heterophoria basically rely on the perception of the client: the “Measurement and correction methodology after H.-J. Haase” (MCH), for example, gives heterophoria measures and corresponding prismatic corrections, under partial dissociation of the two visual inputs (peripheral fusion) and for far viewing distances (6 m; Schroth [29]); participants judge a series of targets and prismatic glasses are used to balance the inputs of both eyes so that targets appear centered around fixation. Note, that (clinically) heterophoria measures typically serve as basis for a prismatic corrections, especially when visual strain is reported [30, 31] and thus, heterophoria is often given in prism-diopter and not in degree of visual angle. This is also true for the next typical method of measuring the heterophoria at close viewing distances (30 cm), i.e. the Maddox Wing test. This simple-to-apply test measures the vergence angle under full dissociation of the two visual inputs (for details see Pointer [32]): participants fixate a scale in one eye and a pointing arrow in the other eye and the perceived position of the arrow on the scale gives the heterophoria measure.

In sum, we expected to replicate binocular advantages in reading fixations by about 10% and attenuating effects of heterophoria. Differential impacts of different, typically used and reported heterophoria measures were additionally explored.

## Methods

### Participants

In total, 102 young volunteers (61 female and 41 male) aged 18 to 40 years (mean 26.3, SD 3.9 years) participated. All participants had an uncorrected visual acuity of 0.8 (in decimal units) or better (6/7.5 equivalent, +0.1 logMAR) at a viewing distance of 60 cm in each eye. All participants were native German speakers. The un-/cover test (to exclude strabismus) and TNO-stereoacuity (60s or better) also showed no obvious strabismic or binocular imbalance. Participants who further showed vertical heterophoria greater than 1 pdpt or who were wearing prismatic corrections were excluded from further data analysis. In sum, only participants with overall good vision and balanced binocular vision were selected for the present sample and thus, finally, data from 94 participants were analyzed.

As part of our orthoptic examination session, all participants were also tested for eye dominance using a sighting test: the participant had to fixate a target (displayed at 5.5 m distance) through a hole (done with both hands at arm length; see Jainta & Jaschinski, [15]). Only 24 of our participants showed a left eye dominance and we replicated a previously reported observation that most people in random samples show a right eye dominance, when tested with sighting tests [33, 34].

### Materials

We recorded the movements of both eyes with the video-based EyeLink II (details provided by SR Research Ltd, Osgoode ON, Canada; sampling frequency 500 Hz). The experimental set up has been used in several previous studies by now and thus, the procedure for presenting targets, calibration, and measuring eye movements is described in details elsewhere [15, 35–37]. In short, horizontal eye movements were recorded for both eyes separately at a viewing distance of 60 cm and calibrations were always run monocularly; for calibration, participants fixated targets that appeared for 1000 ms at one of three horizontal fixation positions (displacement: 8 and 5 deg for reading and heterophoria measures, respectively (see below)). Monocular presentations (right or left eye) were randomly interleaved.

For target presentations we used a mirror stereoscope [5–7] with two half mirrors at right angle and two TFT screens. Both screens were placed at a viewing distance of 60 cm.

For all eye movement measurements, we extracted saccades and fixations using the version signal [(left eye + right eye)/2] and calculated first fixation durations for all words: participants had to read 40 sentences (in total) from the Potsdam-Sentence-Corpus (PSC; see Kliegl, Nuthmann et al. [38]). We selected sentences containing 8 to 13 words, and the sentences differed in total length from 55 to 75-character spaces. Sentences were presented in black, Courier New font size 12, on a white background with a luminance of 24 cd/m2, while the surrounding room lightning was set to about 127 lux.

### Tasks and Procedure

In the binocular reading task participants had to read 20 sentences (which were randomly selected from the total set of 40 German sentences) and they were presented to both eyes simultaneously. All sentences were also presented in 4 blocks of 5 sentences and before each block, we applied a complete calibration run. Between blocks, participants could rest and relax their eyes for a few minutes. The monocular reading task resembled the binocular reading task as described above. However, the sentences were presented to the dominant eye only and represented the other half of the total of 40 German sentences; thus, every sentence was read only once. In 1/3 of the trials participants answered a three-alternative multiple choice question pertaining to the content of the current sentence (responded by mouse click). Participants who showed more than 10 % of incorrect answers in either binocular or monocular reading were excluded from data analysis.

### Measurements of Heterophoria

In total, three different measures of heterophoria were collected and used for further analysis:

1. **Objective heterophoria** was measured according to the method described by Han, Guo et al. [28]: after calibration, the participants fixated a binocular cross for 2.5 s, followed by another cross which was presented to one eye only (for 15 s). Then again, the binocular target was presented for another 2.5 s, followed by a 15 s monocular target to the fellow eye. Binocular recordings (EyeLinkII) were stored all the time and calibrations were run as described above. For all fixation periods (two binocular (2.5 s) and two monocular (15 s)), we calculated the vergence angle and extracted the first interval of 500 ms (binocular) resp. 2000 ms (monocular) close to the end of each fixation period, in which vergence remained stable. Next, for each pair of binocular fixation period followed by a monocular fixation period, we calculated the objective heterophoria as difference between monocular vergence angle at the end of monocular fixation minus binocular vergence angle at the end of binocular fixation. Finally, both measures of objective heterophoria (deg) were averaged for each participant and gave a measure of individual objective heterophoria [28].
2. Heterophoria was also measured with the **Maddox Wing test** (Clement Clarke International Ltd., Harlow, UK) at 30 cm under full dissociation of the visual stimuli [32, 39]. The right eye fixates an arrow, while the left eye fixates a numbered scale. The participant reports where the arrow is observed on the scale. The resulting value is a heterophoria in pdpt.
3. Heterophoria was further measured at a distance of 6 m following the “Guidelines for the application of the **M**easuring and **C**orrecting Methodology after H.-J. **H**aase” (MCH) (see www.ivbs.org for details). **MCH** is a subjective method to measure a patient’s heterophoria at far viewing distances. The targets are presented monocularly under peripheral fusion and prisms are placed before the participant’s eyes until the test objects are aligned. The resulting prism corresponds to the heterophoria in pdpt [29].

Typically, eye movement data (objective heterophoria) are recorded in min arc or degree visual angle and measures obtained in optometric tests (heterophoria with Maddox wing and heterophoria with MCH) are measured in prism diopter (pdpt). To facilitate further analysis all measures objective heterophoria and optometric heterophoria were converted into degrees: optometric heterophoria measures (in pdpt) were multiplied by 0.573 (i.e. arctan (0.01 m/ 1 m)).

### Statistical analysis

In total, data from 94 participants was analyzed: we extracted 190 (± 38) fixations, on average, for reading a set of 20 sentences (one presented binocularly, and one presented monocularly). These observations were pooled within each participant and condition prior to analysis. Next, for statistical data analysis we used a linear mixed-effects model (lmer from package lme4 [40, 41] in R [41]). The statistical package R provides reliable algorithms for mixed effect parameter estimations as well as tools for their evaluation [42]. The p values and confidence intervals were estimated by using posterior distributions for the model parameters obtained by Markov Chain Monte Carlo sampling, including typically a sample size of 10 000 (see for example, Baayen et al. [43]). Predictors were centered, and variables transformed, if necessary.

Presently, our main interest was to compare binocular reading with monocular reading conditions, so reading conditions (monocular vs. binocular) were defined as obvious fixed effect, while participants were treated as random effect. For further analysis the same model was estimated three times, when comparing monocular versus binocular reading and heterophoria measures reflected two additional fixed effects: heterophoria size (S: continuously ranging between 0 and 8) and heterophoria direction (D: eso versus exo).

We will state the estimated fixed effect (b) with its standard error (SE), the t value and the p value.

## Results

**Figure 1:**
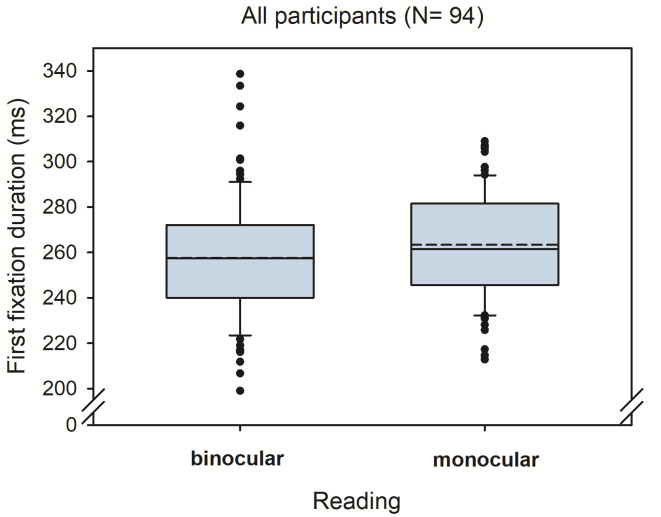
Boxplots for first fixation duration in monocular and binocular reading.

Overall, we found an average binocular advantage of 6 ms (SD = 18) across all 94 participants. Figure 1 shows the corresponding boxplots for monocular and binocular first fixation durations, respectively. The statistical analysis showed a corresponding significant effect for the fixed effect of reading condition (see Table 1).

**Table 1:** Linear-mixed effect models for first fixation duration (ms)

	*b*	*SE*	*t*
(Intercept)	257	2.59	99.36***
Bino / Mono	**6**	1.89	3.03**

Note. *: p ≤ 0.05; **: p ≤ 0.01; ***: p ≤ 0.001.

**Figure 2:**
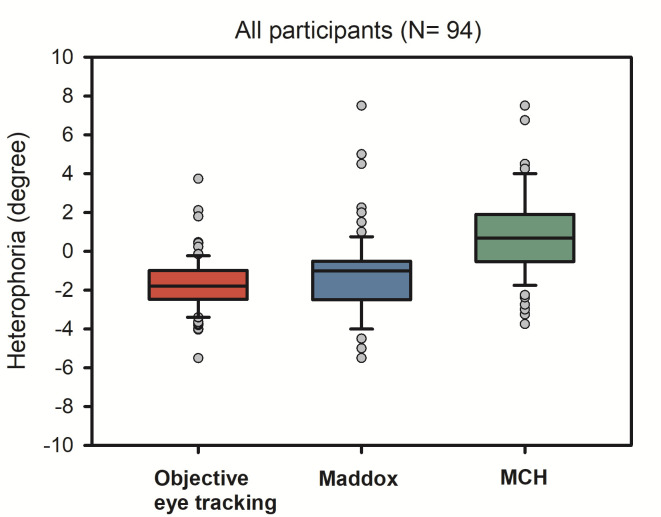
Boxplots of all three heterophoria measurements: objective recordings via EyeLink II (60 cm), Maddox-Wing-Test (30 cm) and MCH (6 m), i.e. Measuring and Correcting Methodology after H.-J. Haase (see above).

**Figure 3:**
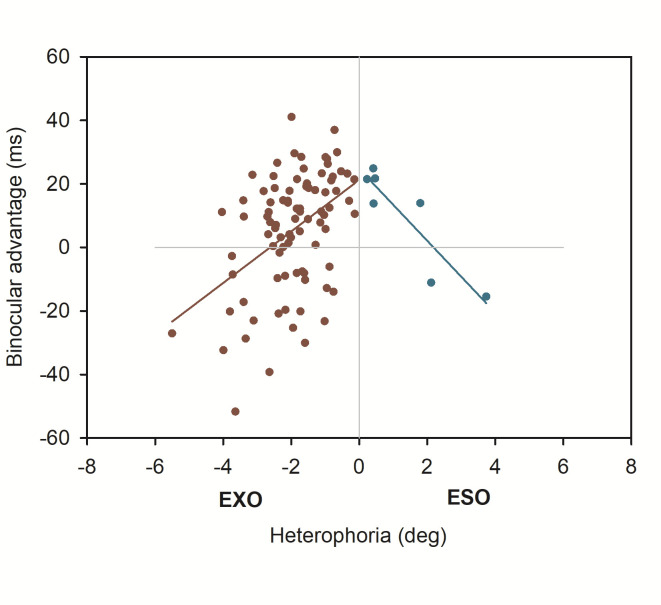
Binocular advantages as function of objective heterophoria (EyeLink II).

Heterophoria measurements showed substantial exo- and esophorias, ranging from -6 degrees (exo) to 8 degrees, (eso), respectively. Figure 2 shows a boxplot of all three heterophoria measurements. Next, we calculated linear mixed effect models including heterophoria measures (see Table 2): when objective eye tracking measures of heterophoria were included, overall estimation of the binocular advantage increased; this advantage showed a significant dependence on heterophoria size (i.e. the interaction: reading condition x heterophoria size was significant), reflecting smaller binocular advantages when heterophoria increased. Heterophoria direction (exo versus eso) showed no statistical effect on the binocular advantage and the three-way-interaction did not add any information as well. Figure 3 shows the change in binocular advantage with increasing heterophorias in eso and exo direction for objective eye tracking heterophoria measures.

**Table 2:** Linear-mixed effect models for first fixation duration (ms), dependent on monocular versus binocular reading (M) and heterophoria size (S) and heterophoria direction (D: eso versus exo); in (a) objective measures were analysed, in (b) Maddox-Wing measures and in (c) MCH measures of heterophoria.

*(a)*	*b*	*SE*	*t*
(Intercept)	241	13.98	17.27***
M: Bino/Mono	**25**	9.13	2.71**
S: Het Size	5	7.83	0.69
D: Het Direction	7	15.15	0.46
M x S	**-11**	5.11	-2.21*
M x D	-4	9.90	-0.36
S x D	-1	8.27	-0.04
M x S x D	3	5.40	0.60
			
*(b)*	*b*	*SE*	*t*
(Intercept)	254	7.01	36.25***
M: Bino/Mono	**20**	4.88	4.03**
S: Het Size	-1	2.83	-0.31
D: Het Direction	2	8.51	0.23
M x S	**-5**	5.11	-2.46*
M x D	**-12**	9.90	-1.99*
S x D	2	8.27	0.60
M x S x D	3	5.40	1.08
			
*(c)*	*b*	*SE*	*t*
(Intercept)	254	4.89	51.88***
M: Bino/Mono	**9**	3.54	2.73**
S: Het Size	1	1.75	0.20
D: Het Direction	1	8.41	0.18
M x S	-1	1.27	-0.85*
M x D	-5	6.18	-0.78*
S x D	5	4.69	1.14
M x S x D	-1	3.46	-0.17

Note. *: p ≤ 0.05; **: p ≤ 0.01; ***: p ≤ 0.001.

**Figure 4:**
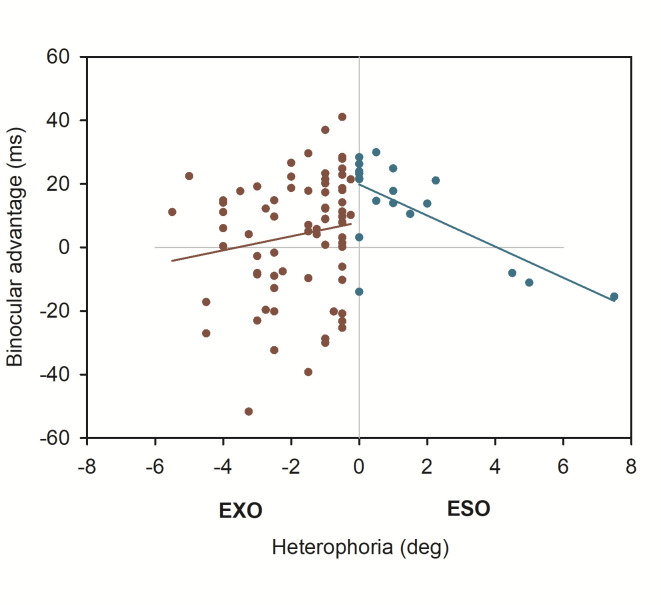
Binocular advantages as function of Maddox-Wing heterophoria.

As displayed in Table 2, the linear mixed effect model showed slightly different parameter estimations, when Maddox-Wing measures of heterophoria were included: overall estimation of the binocular advantage increased again, and this advantage again decreased with increasing heterophoria. But this time heterophoria direction (exo versus eso) showed a statistical effect on binocular advantages, i.e. exophorias reduced binocular advantages significantly when compared to esophorias. The three-way-interaction did not add further any information and Figure 4 shows the change in binocular advantage with increasing heterophorias in eso and exo direction for Maddox-Wing measures.

**Figure 5:**
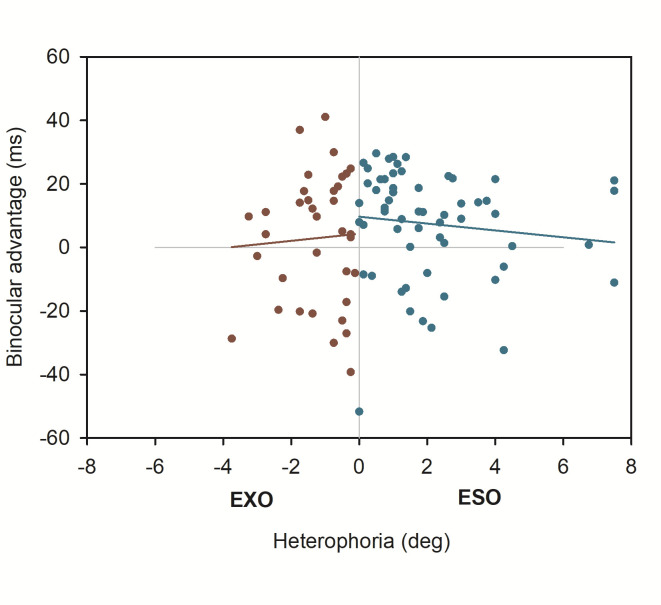
Binocular advantages as function of MCH heterophoria.

Further, as displayed in Table 2, the linear mixed effect model showed again different parameter estimations, when MCH measures of heterophoria were included: overall estimation of the binocular advantage were small, almost as small as estimated by the first model without heterophoria measures included, and no effect of heterophoria showed statistical significance (see also Figure 5 for a display of binocular advantages in reading over MCH heterophoria measures).

### Analysis for an orthophoric subsample

**Figure 6:**
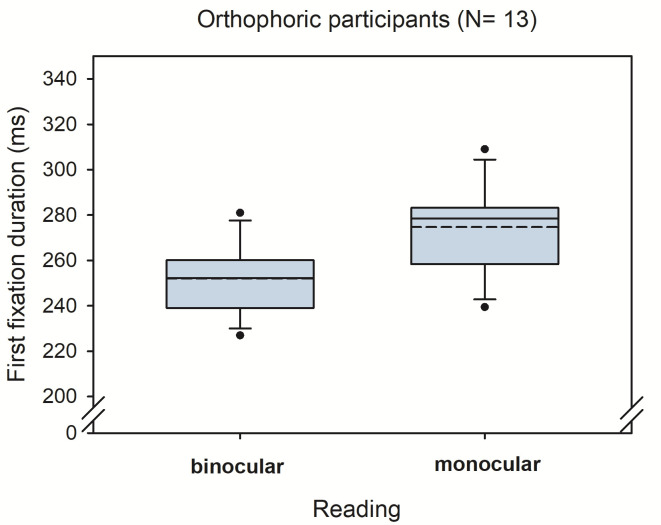
Boxplots for first fixation duration in monocular and binocular reading for orthophoric participants only.

Only few participants could be characterized as being orthophoric: 13 participants showed heterophorias smaller than ± 0.5 degrees in all three tests. For these orthophorics the average binocular advantage was 23 ms (SD = 7), on average, and thus, higher compared to the total sample and close to the estimates of binocular advantages when objective and Maddox heterophorias were accounted for (see Figure 6 and Table 3).

**Table 3:** Linear-mixed effect models for first fixation duration (ms) and orthophoric participants only.

	*b*	*SE*	*t*
(Intercept)	252	4.84	52.01***
Bino / Mono	**23**	1.83	12.30***

Note. *: p ≤ 0.05; **: p ≤ 0.01; ***: p ≤ 0.001

## Discussion

In the present study, we replicated binocular advantages (shorter fixation times) of about 20 ms in reading, i.e. binocular advantages of about 10% [1–3, 11, 15]. Further, individual aspects of motor fusion (i.e. heterophoria) impacted on reading efficiency and reduced binocular advantages when heterophoria was measured objectively via eye tracking methods or the Maddox-Wing-Test.

One striking result in the present study is, that effect estimations of binocular advantages were much lower for the total sample when heterophoria was not accounted for (less than 5 %); as soon as heterophoria measures (tested objectively via eye tracking methods or the Maddox-Wing-Test) were included in estimations of binocular advantages, values of about 20 ms for first fixation durations emerged constantly. These 20 ms were nicely in line with the effect estimations done for orthophoric participants, i.e. for participants that showed no heterophoria (in all three tests) at all and with previous reports, respectively [1–3, 11, 15]. Such results are further in agreement with reports of binocular advantages as a consequence (and summation) of several visual functions [2, 5–7, 16] and thus, are also present in a complex, dynamic task like reading. In other words, effective binocular vision critically enriches the delivery of visual information necessary for efficient reading.

Moreover, the exploration of different, clinically used methods of measuring heterophoria also yield interesting results: while tracking the eye objectively with an eye tracker and extracting heterophoria from these signals [28] gave an obvious impact of heterophoria size on binocular advantages, the two other subjective methods gave a different pattern. But let’s start with the objective measurements of heterophoria: we found a diminishing effect of heterophoria size, i.e. reduction by half for each additional degree of heterophoria, regardless of the direction of heterophoria. This reflects a very balanced effect for esophoria and exophoria and corresponds to previous observations for exophoria only [15]. In the latter case, we showed, that all parameters of binocular coordination (fixation disparity, vergence adjustments during fixations, saccade disconjugacy) changed dramatically, while fixation duration decreased in monocular reading; in other words, when bin-ocular fusion was disabled during monocular reading for this subgroup, no oculomotor adjustments were needed and processing the visual input was relatively fast. But as soon as binocular fusion processes were enabled under binocular reading, these oculomotor adjustments needed time to best overlap both visual inputs and, as a consequence, prolonged reading fixations for readers with distant resting states of vergence (i.e. large exophorias; Jainta and Jaschinski [15]). This argumentation might also hold for the present data set: the larger the exo- or esophoria, the higher the need for fine-tuned binocular coordination during binocular reading and the smaller the benefit compared to monocular reading. Note that objective measurements of heterophoria were taken at 60 cm viewing distance, i.e. the reading distance.

It is also important to note, that all horizontal heterophoria methods in this study are only used to indicate individual heterophoria; heterophoria is defined here as the vergence angle that results from proximal aspects, tonic vergence and accommodation; it is used as well-established parameter to indicate the resting state of the vergence system [9, 17, 20]. Neither corrections nor reductions in asthenopia or eye symptoms were the focus of the present study (actually, only few participants reported eye or reading related symptoms or asthenopic problems). Further, all optometric heterophoria test are used in their typically used version, that is, the Maddox wing test at 30 cm distance and the MCH test at 6 m. We are aware of the fact, that all tests could be rescaled for different distances, but this is not the typical – and clinical – use. Please also note, that all optometric tests for horizontal heterophoria are typically used for extrapolations to reading situations. Therefore, rescaling all optometric tests might not help in addressing relations to reading behavior, as long as such rescaling is not part of the day-to-day routines in clinical practice.

Taking into account, that all used optometric tests are applied at difference viewing distances, the pattern of our results showed interesting changes when measures of subjective heterophoria were considered: when heterophoria was measured using the Maddox-Wing-test at 30 cm viewing distance [32], heterophoria size marginally reduced the binocular advantage again but this time, exophoric participants showed overall lower binocular advantages in general. Since the Maddox-Wing measures are reported to show sufficient reliability [32, 39], we could only speculate why the impacts of tested heterophoria on binocular advantages looked differently; maybe the typically used set-up for Maddox-Wing tests do not give optimal measures to generalize to reading at different viewing distances.

The same is true for MCH heterophoria measures: our data shows no obvious relation to binocular advantages during reading. The MCH (Measuring and Correcting Methodology after H.-J. Haase) gives heterophoria measures at far viewing distances (6 m) and gives typically basic estimations for prismatic corrections [29]. The MCH is also the only method which used targets including peripheral fusion locks [5–7]. Thus, vergence did not “float” in a “open-loop” status and since all targets are presented at 6 m, accommodation did not contribute to the heterophoria as well [5–9, 31]. These differences in testing set-up clearly separates the MCH heterophoria measure from the two other measures, namely, the objective measure and the Maddox-Wing test. Further research is clearly needed to explore and evaluate the usefulness and impact of different heterophoria measure as indicators of binocular advantages and eye movement behavior in several, day-to-day tasks.

## Conclusion

Binocular advantages in reading when quantified by first fixation duration on words amount to about 10% and individual heterophoria reduces this effect by about half per degree of increased eso- or exophoria. This impact of individual horizontal heterophoria could best be estimated by objective eye tracking measures of heterophoria, which were collected at reading distance.

## Ethics and Conflict of Interest

Each subject gave written informed consent before the experiments; the research followed the tenets of the Declaration of Helsinki and was approved by the Swiss ethics committee (https://www.swissethics.ch/; Project ID: 2017-01155).
